# Downstream Transport of Geosmin Based on Harmful Cyanobacterial Outbreak Upstream in a Reservoir Cascade

**DOI:** 10.3390/ijerph19159294

**Published:** 2022-07-29

**Authors:** Jae-Ki Shin, Yongeun Park, Nan-Young Kim, Soon-Jin Hwang

**Affiliations:** 1 Busan Region Branch Office of the Nakdong River, Korea Water Resources Corporation (K-water), Busan 49300, Korea; shinjaeki@gmail.com; 2 School of Civil and Environmental Engineering, Konkuk University, Seoul 05029, Korea; yepark@konkuk.ac.kr; 3 Department of Environmental Health Science, Konkuk University, Seoul 05029, Korea; celeste0@hanmail.net

**Keywords:** *Dolichospermum circinale*, geosmin, hydrodynamics, hydropower release and tailwater, multiple-impoundment series

## Abstract

Understanding water quality events in a multiple-impoundment series is important but seldom presented comprehensively. Therefore, this study was conducted to systematically understand the explosion event of geosmin (GSM) in the North Han River (Chuncheon, Soyang, Euiam, and Cheongpyeong Reservoirs) and Han River (Paldang Reservoir), which consists of a cascade reservoir series, the largest drinking water source system in South Korea. We investigated the spatiotemporal relationship of harmful cyanobacterial blooms in the upstream reservoir (Euiam) with the water quality incident event caused by the GSM in the downstream reservoir (Paldang) from January to December 2011. The harmful cyanobacterial bloom occurred during August–September under a high water temperature (>20 °C) after a heavy-rainfall-based flood runoff event. The high chlorophyll-*a* (Chl-*a*) concentration in the upper Euiam Reservoir was prolonged for two months with a maximum concentration of 1150.5 mg m^−3^, in which the filamentous *Dolichospermum circinale* Kütz dominated the algal community at a rate of >99%. These parameters remarkably decreased (17.3 mg Chl-*a* m^−3^) in October 2011 when the water temperature decreased (5 °C) and soluble reactive phosphorus was depleted. However, high and unprecedented GSM concentrations, with a maximum value of 1640 ng L^−1^, were detected in the downstream reservoirs (Cheongpyeong and Paldang); the level was 11 times higher than the value (10 ng L^−1^) recommended by the World Health Organization. The concentrations of GSM gradually decreased and had an adverse effect on the drinking water quality until the end of December 2011. Our study indicated that the time lag between the summer–fall cyanobacterial outbreak in the upstream reservoir and winter GSM explosion events in the downstream reservoirs could be attributed to the transport and release of GSM through the effluent from hydroelectric power generation in this multiple-reservoir system. Therefore, we suggest that a structural understanding of the reservoir cascade be considered during water quality management of drinking water sources to avoid such incidents in the future.

## 1. Introduction

Off-flavor substances, such as geosmin (GSM), in freshwater can result in an earthy odor, particularly in drinking water resources [1], which can be detected by humans even at very low concentrations (approximately below 30 ng L^−^^1^) [2,3]. The biochemical structure and properties of GSM in freshwater have been explored over the years [4,5,6] and the causative organisms that release GSM have also been extensively studied [7,8,9,10,11]. GSM has emerged as a research subject in rivers and reservoirs used as drinking water sources because it induces unwanted odors in tap water [7]. These odorous substances often occur in high concentrations at specific times and areas [9].

The closely-related relationship between filamentous cyanobacteria (e.g., *Dolichospermum* population) and GSM has been actively studied. The outbreak of *Dolichospermum* (formerly *Anabaena*) is related to various environmental factors, particularly water temperature [10,12], nutrients [13,14], summer heat waves [15], and low-flow conditions [16]. Conversely, this abundant phytoplankton is primarily transported downstream by high-flow events or their growth is inhibited, but they can also occur and perish within the reservoir during long-term low-flow events. Although few or no direct risks are known to date, even a very small amount (<10 ng L^−1^) of GSM is expressed as a representative odorant that stimulates the human olfactory system [7,17,18,19]. This deteriorates the water quality, even resulting in public complaints and countermeasures. Moreover, this acts as a social risk factor by aggravating the economic burden and inconvenience in daily life activities [3,19,20,21,22,23,24,25,26].

Generally, the construction of dams and subsequent impoundment of rivers result in altered hydrology, increased retention time, and associated changes in water quality [27]. In contrast, these dams contribute positively to daily life and socioeconomic aspects but lead to significant environmental damage [28]. In most cases, each river has one dam reservoir; in other cases, the flow rate is controlled by several cascade dams such that the hydraulic head is actively utilized [29]. A multiple-reservoir series can efficiently utilize limited water resources through hydroelectric power generation. However, if a reservoir located upstream is vulnerable to eutrophication (i.e., harmful algal blooms), the water quality of the downstream reservoir deteriorates [13]. Reservoir eutrophication can increase the quantity of a single or several species of phytoplankton, as various external and internal factors increase the amount of nutrients in the water, leading to outbreaks of undesired harmful cyanobacteria that produce toxins or affect the taste and odor of water [30]. Thus, water quality patterns within and between multiple-reservoir series are spatiotemporally more complicated [31].

Multiple reservoirs along a river produce unique gradients of reservoir water quality [32]. Walker [33] developed empirical methods to predict eutrophication in impoundments using surface total phosphorus, Secchi depth, and chlorophyll-*a* (Chl-*a*) values in terms of Carlson’s [34] trophic state indices for four reservoirs in the White River system in the USA. De Oliveria Naliato et al. [31] demonstrated that the discharge pulses of cascade hydropower dams are important in such investigations to verify whether high-amplitude and low-frequency variations can negatively affect the downstream river biota for two reservoirs in the Paranapanema River in southeastern Brazil. Accordingly, the limnological conditions of the upstream and downstream reservoirs have been frequently compared. Few studies have explored the relationship between cyanobacteria and GSM behavior in a reservoir cascade compared with that in single reservoirs. In the multiple-reservoir series located in the North Han River water system in South Korea, toxic (e.g., *Dolichospermum circinale*: Microcystin-LR 3.58–5.22 × 10^−4^ pg cell^−1^, Microcystin-RR 6.47–10.31 × 10^−4^ pg cell^−1^; J.-K. Shin, unpublished data) and nontoxic harmful cyanobacteria have repeatedly flourished almost every year since 1986. The highest recorded levels of Chl-*a* concentration were 4787.0 mg m^−3^ in August 2010 and 1115.5 mg m^−3^ in September 2011 in the upstream Euiam Reservoir. However, the GSM events during 2010 and 2011 were completely different. The high GSM concentration in the downstream reservoir caused an incident in the early winter of 2011 that temporarily cut off the domestic water supply from this reservoir.

Thus, the purpose of this study was to understand the spatiotemporal dynamics of GSM based on harmful cyanobacterial blooms in a unique reservoir cascade in South Korea. Our results suggest the importance of monitoring the subsequent water quality impact caused by harmful cyanobacteria associated with hydropower-related effluents from reservoirs with continuously occurring harmful cyanobacteria because of increased eutrophication.

## 2. Materials and Methods

### 2.1. Description of the Study Site

**Figure 1:**
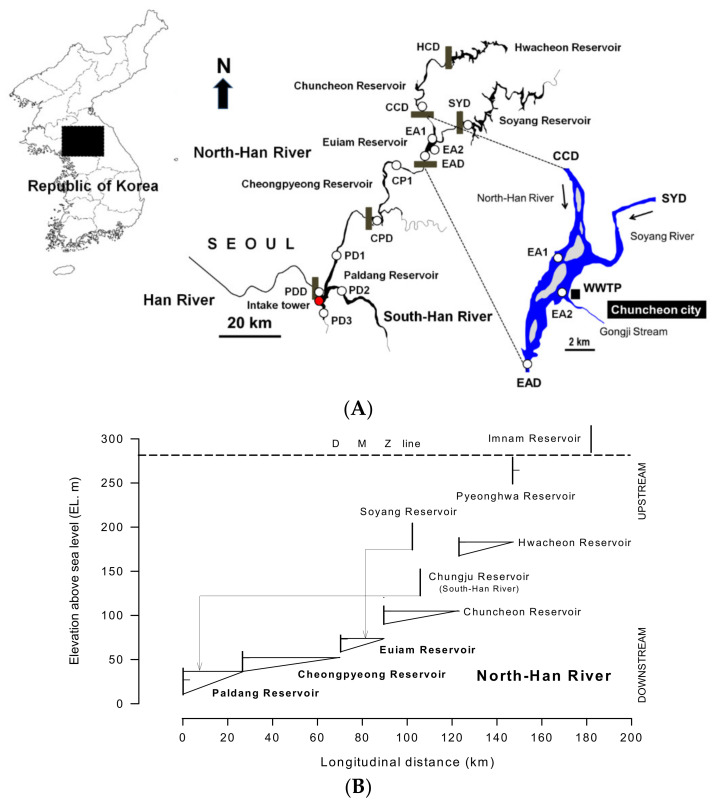
(**A**) Map and (**B**) schematic diagram showing the sampling stations and target study area (Euiam–Paldang Reservoirs) located in the North Han River and Han River system. Field study and data used were from January to December 2011. The EA2–EAD stations in Euiam Reservoir were additionally surveyed on 13 August and 19 September 2011. HCD, Hwacheon Reservoir dam; CCD, Chuncheon Reservoir dam; SYD, Soyang Reservoir dam; EAD, Euiam Reservoir dam; CPD, Cheongpyeong Reservoir dam; PDD, Paldang Reservoir dam; WWTP, wastewater treatment plant.

The North Han River (37°52′657″ N–38°11′731″ N, 127°27′917″ E–127°77′898″ E) is the first tributary of the Han River, the largest in South Korea, and a transboundary-shared river with its source and upstream connected to North Korea. It is a multiple-reservoir series (the only one in Korea) with five reservoirs connected in a cascade from upstream: Hwacheon (38°11′731″ N, 127°77′898″ E), Chuncheon (37°96′866″ N, 127°66′847″ E), Euiam (37°83′609″ N, 127°67′599″ E), Cheongpyeong (37°70′909″ N, 127°45′549″ E), and Paldang (37°52′657″ N, 127°27′917″ E) (Figure 1). Soyang Reservoir (29.0 × 10^9^ m^3^), which is the largest reservoir in South Korea, is located in a tributary (Soyang River) of the Euiam Reservoir (Figure 1). Table 1 provides a summary of the main specifications and properties of the reservoirs. Among these, only Hwacheon and Soyang have storage capacities > 10 × 10^9^ m^3^; whereas those of the other reservoirs range from 0.8 to 2.4 × 10^9^ m^3^ (Figure 1 and Supplementary Table S1). The water storage volume in the middle and lower reaches of the North Han River and Han River section increased towards the downstream region (Euiam < Cheongpyeong < Paldang). Simultaneously, the water depth, water surface width, and longitudinal reservoir length also increased according to the scale of water storage.

The Euiam Reservoir is unique in that it is adjacent to a relatively large city (Chuncheon, Gangwon Province) in the North Han River watershed. Since the initial impoundment in 1968, severe blooms caused by harmful cyanobacteria (*Dolichospermum* *circinale*) and their negative impacts have continuously persisted in the Gongji Stream river mouth near the discharge port for the treated wastewater of the Chuncheon wastewater treatment plant (WWTP) [35,36,37,38,39]. Consequently, a potential exists for GSM production that can affect not only Euiam Reservoir but also the downstream Cheongpyeong and Paldang Reservoirs, depending on the occurrence pattern of *D. circinale* [37,40,41,42].

In addition to small-scale agriculture and drinking water, the large artificial dam reservoir in South Korea is used for several purposes, such as flood control and water supply, and it functions as a hydroelectric power source [27,29,43]. Therefore, water is discharged through hydroelectric power outlets installed in the middle or lower layers of such reservoirs, except during flood events [38,43]. The Paldang Reservoir in the Han River is the largest water source that supplies domestic and industrial water to the metropolitan area (Seoul, Gyeonggi Province). Except for the short-term water gates opening during flooding, most of the river water is dependent on hydrological control through hydropower-related discharge of the North Han River (Cheongpyeong Reservoir) and South Han River (Chungju Reservoir, 37°00′629″ N, 127°99′260″ E).

### 2.2. Acquisition of Data on Rainfall, Hydrological Factors, and Water Quality

Rainfall and hydrological data (inflow, discharge, and water level) measured in the watersheds of the Hwacheon–Cheongpyeong Reservoirs of the North Han River and Paldang Reservoir of the Han River were obtained from the Korea Meteorological Administration (http://www.kma.go.kr (accessed on 1 January 2012)) of the Ministry of Environment (MOE), Republic of Korea, and the National Water Resources Management Information System (http://www.wamis.go.kr (accessed on 1 January 2012)) of the Ministry of Land, Infrastructure, and Transport, respectively. In addition, reservoir water quality data, measured and uploaded monthly, were obtained from the MOE. Considering data availability, 11 sites of the water quality monitoring network were selected: the Chuncheon Reservoir (CCD) of the North Han River and Soyang Reservoir (SYD) of the Soyang River each had one site near the dam; whereas the Euiam Reservoir (EA1, EA2, and EAD) and Cheongpyeong Reservoir (CP1 and CPD) of the North Han River and the Paldang Reservoir (PD1, PD2, PD3, and PDD) of the Han River each had two to four sites (Figure 1 and Table 1). Water quality measurement data produced at the WWTP, a point source of pollution, were also obtained from the MOE.

### 2.3. Field Survey and Water Quality Analysis

One site between EA2 and EAD (EA2–EAD) and a layer at 4 m depth in EAD with prolonged harmful cyanobacterial bloom in the Euiam Reservoir were investigated on 13 August and 19 September 2011, respectively. The water quality, phytoplankton cell count, and GSM concentration at EAD, CPD, PD1, and PDD were monitored daily from 28 November 2011 to 4 January 2012. A field survey was conducted at each site. Water temperature and dissolved oxygen (DO) were measured using a YSI-550A (YSI Inc., Yellow Springs, OH, USA), pH was measured using an Orion-230A (Thermo Scientific, Waltham, MA, USA), and conductivity was measured using a WTW-33 meter (Cole-Parmer, Vernon Hills, IL, USA). The samples for various water quality analyses were collected within 0.2 m depth from the water surface using a horizontal Van Dorn sampler (Wildcore, Norwich, CT, USA). The collected samples were placed in an icebox, transferred to the laboratory within 12 h, and immediately pretreated and analyzed [44]. Total nitrogen (TN) and total phosphorus (TP) were analyzed using raw water following standard methods [44]. The samples for the analysis of dissolved nutrients (dissolved TN, dissolved TP, ammonium (NH_4_), nitrate (NO_3_), and soluble reactive phosphorus (PO_4_)) were filtered using a Whatman GF/F filter (Whatman, UK) [44,45]. The Chl-*a* concentration was analyzed by the boiling extraction method using 90% ethanol (Sigma-Aldrich, Steinheim am Albuch, Germany) after filtering an appropriate amount of sample through a Whatman GF/F filter [46]. Samples for phytoplankton observation were prepared immediately after collection with Lugol’s solution (Sigma-Aldrich, Steinheim am Albuch, Germany), and samples concentrated without disturbance for one week in the dark were counted using a Sedgewick Rafter counting cell slide (SPI, Washington, DC, USA). The number of cells (cells mL^−1^) and relative abundance (%) were then calculated [44]. The taxonomic composition of the phytoplankton was identified based on the methods used by Hirose et al. [47] and Prescott [48].

GSM in water was analyzed using gas chromatography/mass spectrometry (GC/MS) according to the headspace solid-phase microextraction (HS-SPME) method [49]. The assay sample (1 mL) was prepared and transferred to a 15 mL vial (15 mL clear vial, screw-top hole cap with PTFE/Silicone Septa; Supelco, Bellefonte, PA, USA) using a magnetic bar. The vials were incubated in a heating block at 40 °C for 30 min with continuous stirring at 400 rpm on a hotplate stirrer to promote the accumulation of gaseous compounds in the vial headspace. A polydimethylsiloxane-coated fiber (Stableflex 2 cm SPME Fiber PK3, 50/30 µm DVB/Carboxen; Supelco, Bellefonte, PA, USA) was inserted into the vial headspace until the volatile organic compounds reached absorption equilibrium. After extraction, the fiber containing the absorbed compounds was inserted into a GC injector at 250 °C for 5 min for thermal desorption. An HP-5MS (Agilent Technologies, Santa Clara, CA, USA) column was used for GC/SPME analysis through GC (GC-2010 Plus; Shimadzu, Kyoto, Japan). The solid-state GSM (16423-19-1; Sigma-Aldrich, Burlington, MA, USA) was dissolved in methanol to obtain a 10 g L^−1^ standard solution. Standard curves were generated for OC quantitative analysis by serially diluting the standard with distilled water and then measuring the concentration using the GC/SPME method [49]. The GSM standard was detected at a 17.53 min retention time.

### 2.4. Descriptive Statistical Analysis

We used analysis of variance post-analysis (post hoc Tukey’s honest significant difference test) to analyze spatiotemporal differences between sites, and performed correlation analysis between factors using Spearman’s rank analysis. The data were statistically processed using SYSTAT^®^ 8.0 (SPSS Inc., Chigaco, IL, USA) [50], and significance was set at α = 0.05.

## 3. Results

### 3.1. Characteristics of the Main Hydrometeorological Factors

Table 1 summarizes the descriptive statistics using daily data of rainfall, inflow and outflow discharges, and water levels observed at dam reservoirs located in the North Han River and Han River systems from January to December 2011. The inflow and outflow increased from upstream to downstream (Table 2) (*p* < 0.001). From June to December 2011, the fluctuations in inflow and outflow showed an almost linear relationship (*r* = 0.999, *p* < 0.001), and the reservoir size depended on the amount of water stored (Euiam < Cheongpyeong < Paldang). The Cheongpyeong and Paldang Reservoirs were larger than the Euiam Reservoir because of the confluence of the large tributaries of the Hongcheon and South Han Rivers, respectively. Additionally, the fluctuation range of the Paldang Reservoir in terms of inflow and water level was small at 1.1 m, whereas those of the Euiam and Cheongpyeong Reservoirs were slightly larger at 1.7 and 2.0 m, respectively (Table 2). The discharges of Chuncheon Reservoir (*r* = 0.992, *p* < 0.01) and Soyang Reservoir (*r* = 0.939, *p* < 0.01) showed a strong positive correlation with the flow rate variability of the Euiam Reservoir. The discharge of Euiam was positively correlated with the flow rate variability of Cheongpyeong (*r* = 0.970, *p* < 0.01), and that of Cheongpyeong (*r* = 0.882, *p* < 0.01) was positively correlated with the flow rate variability of Paldang. In addition, the degree of the relationship tended to decrease slightly from upstream to downstream.

**Figure 2:**
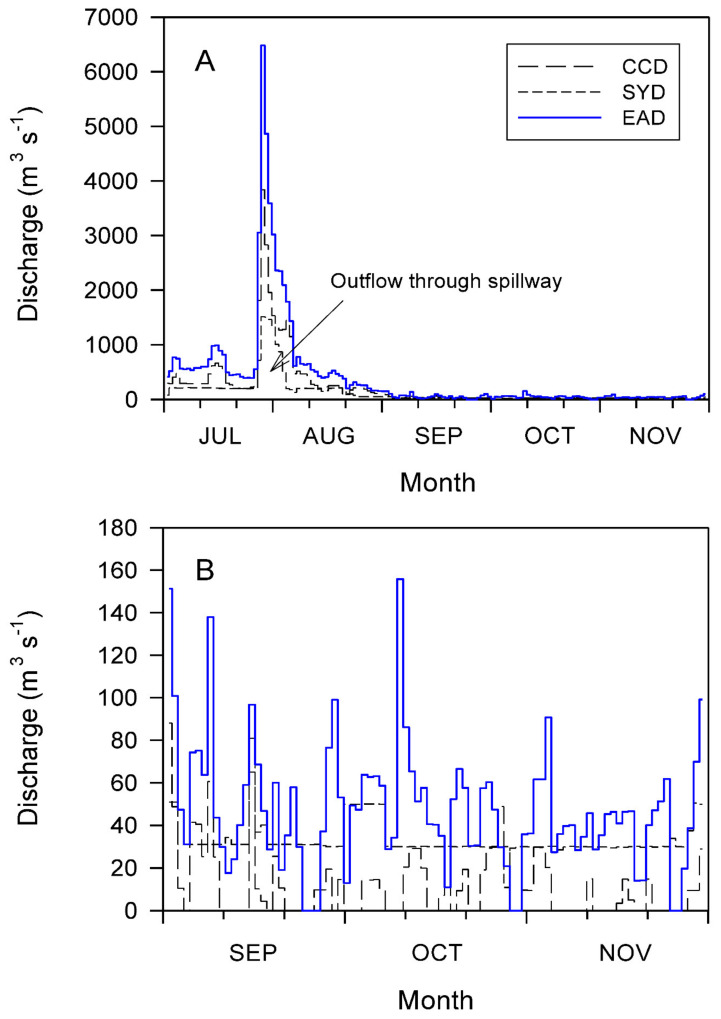
(**A**) Daily fluctuations in the outflow of the Chuncheon, Soyang, and Euiam Reservoirs (CCD, SYD, and EAD, respectively) of the North Han River from July to November 2011. (**B**) Expanded view from September to November 2011.

Figure 2A shows the fluctuations in the daily discharges of the Chuncheon, Soyang, and Euiam Reservoirs from July to November 2011. A long-term simultaneous discharge through the spillway was observed from the multiple-reservoir series between 27 July and 5 August 2011. The total discharge volumes during this period were 18,496.2 m^3^ s^−1^ from the Chuncheon Reservoir, 9310.9 m^3^ s^−1^ from Soyang, and 31,035.2 m^3^ s^−1^ from Euiam. The Euiam Reservoir underwent a very large hydrological change with a retention time of 0.1 d^−1^ based on the water storage volume (80.0 × 10^6^ m^3^). Subsequently, the flow rate gradually decreased from late August to early September (Figure 2A). From September to November 2011, the average discharge volumes of the Chuncheon, Soyang, and Euiam Reservoirs were 11.9, 33.3, and 47.3 m^3^ s^−1^, respectively (Figure 2A,B). A high hydropower discharge of 49.9 m^3^ s^−1^ occurred from the Soyang Reservoir in mid-September, which was weak compared with those from the Euiam and Cheongpyeong Reservoirs in early October (121.6 and 119.1 m^3^ s^−1^, respectively) (Figure 2B). Moreover, a small pulse flow was observed from the downstream Paldang Reservoir at the end of November 2011. However, 44 and 7 d with no discharge were recorded in the Chuncheon and Euiam Reservoirs, respectively (Figure 2A).

**Figure 3:**
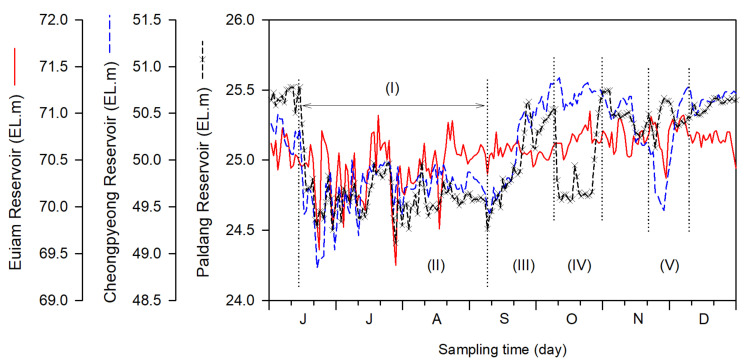
Comparison of daily water-level fluctuations (WLFs) in the Euiam and Cheongpyeong Reservoirs of the North Han River and Paldang Reservoir of the Han River from June to December 2011. The spillway discharging periods of the three reservoirs occurred simultaneously from 27 July to 5 August 2011. (I) Impound and discharge (II) Bloom potential (III) Proliferation of cyanobacterial bloom (IV) 1st transport and dispersion to Cheongpyeong Reservoir (V) 2nd transport and dispersion to Paldang Reservoir.

These results were also reflected in the water-level fluctuations (WLFs). The flow rate and water level showed a significant negative correlation (*r* = −0.317 to −0.544, *p* < 0.01). The WLFs were large (19.8–29.0 m) in the Hwacheon and Soyang Reservoirs, which have high water storage capacities, whereas those of the Chuncheon, Euiam, Cheongpyeong, and Paldang Reservoirs were small (1.1–2.1 m), showing a slight difference depending on the reservoir (*p* < 0.001). After the rainy season, the water level in Euiam continued to increase until the end of October 2011 (EL. 71.1 m) and then increased or decreased repeatedly until the end of November. The Cheongpyeong Reservoir maintained a high water level until early November, after which it decreased significantly (EL. 49.5 m) until the end of November 2011 (Figure 3).

### 3.2. Annual Variations of TN and TP in Major Pollution Sources

**Figure 4:**
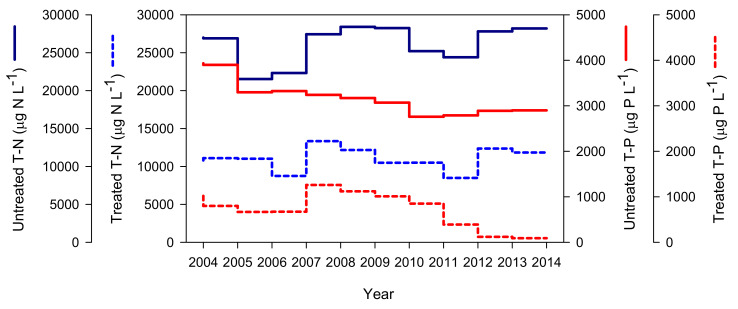
Comparison of the annual mean total nitrogen (TN) and total phosphorus (TP) concentrations in the untreated and treated wastewaters of the Chuncheon wastewater treatment plant located in the Euiam Reservoir watershed from 2004 to 2014. Chemical treatments to mitigate the total phosphorus load began in 2011 to 2012. Data were collected from the Annual Report of National Wastewater Statistics published by the Ministry of Environment (MOE), Republic of Korea.

Figure 4 shows the distributions of the annual average TN and TP concentrations of untreated and treated wastewater discharged from 2004 to 2014 from the Chuncheon WWTP (treatment capacity: 150 × 10^3^ m^3^ d^−1^), which discharges treated wastewater near the Gongji Stream river mouth as the largest source of water pollution in the Euiam Reservoir Basin. In this WWTP, the N and P treatment efficiencies (outflow/inflow concentration × 100) were 57.9% and 77.6%, respectively. The average TN concentrations (treatment rate) of the WWTP effluent were 11,021.3 µg N L^−1^ (57.3%) and 10,896.7 µg N L^−1^ (59.6%) before and after 2011, respectively, showing no significant difference. Conversely, the TP significantly decreased from 925.0 µg P L^−1^ (71.9%) to 200.0 µg P L^−1^ (92.9%). Only the TP concentration decreased significantly in 2014 (Figure 4). In particular, the TP increased slightly from an average concentration of 829.0 µg P L^−1^ in 2004–2006 to 982.6 µg P L^−1^ in 2007–2011 (1130 µg P L^−1^ during 2008–2010) (Figure 4).

### 3.3. Monthly Variations of the Chl-a Concentration

**Figure 5:**
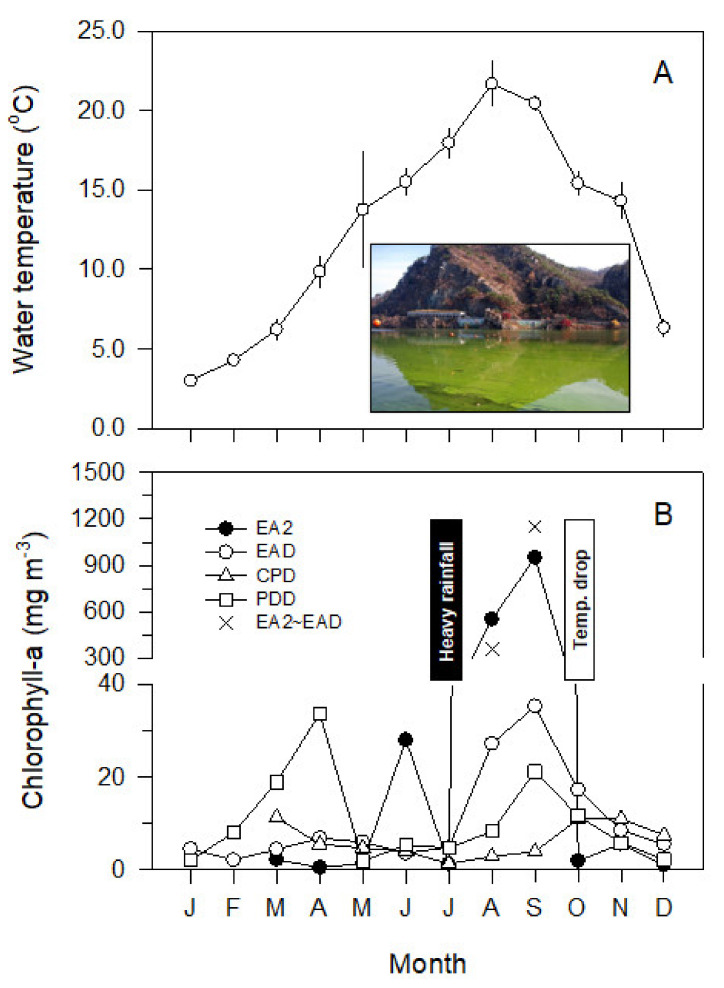
Spatial and temporal variations of the (**A**) chlorophyll-*a* concentration and (**B**) water temperature in the Euiam and Cheongpyeong Reservoirs (EAD and CPD, respectively) of the North Han River and Paldang Reservoir (PDD) of the Han River from January to December 2011. The inset photo shows massive harmful cyanobacterial bloom at the EA2–EAD site of Euiam Reservoir in September 2011.

Table 2 summarizes the water quality factors and measured values for each site in the multiple-reservoir series. The basic water quality primarily varied because of the seasonality or influence of pollutants (e.g., the Gongji Stream, Chuncheon WWTP). The spatiotemporal difference in nutrients in the water was remarkable for TN and NO_3_ (*p* < 0.05) (Table 2). Figure 5 shows the spatiotemporal distribution of the Chl-*a* concentration. At the EA2 site, the Chl-*a* concentration ranged from 0.5 to 950.8 mg m^−3^, and the average value was 154.3 mg m^−3^. The average value from January to May 2011 was 1.3 mg m^−3^, which temporarily increased to 28.0 mg m^−3^ in June and decreased sharply to 1.2 mg m^−3^ in July (Figure 5). Subsequently, it increased exponentially to 550.7 mg m^−3^ in August and 950.8 mg m^−3^ (>99% relative abundance of *D. circinale*) in September, and decreased sharply again from October to December 2011, to show an average value of 2.8 mg m^−3^ (Figure 5). At the EAD site, the average value of the Chl-*a* concentration in the surface layer was 26.6 mg m^−3^ from August to October. The concentrations were very high at the site between EA2 and EAD (356.5 mg m^−3^ in August and 1150.5 mg m^−3^ in September), and the high-density accumulation of *D. circinale* was remarkable in the vicinity of these sites (Figure 5). However, the Chl-*a* concentration in the layer of the EAD site below 4 m was considerably lower than that in the surface layer, and was less than 3.0 mg m^−3^. Notably, the mean value in the CPD site was 6.3 mg m^−3^. Additionally, the average value at the PDD site was 24.6 mg m^−3^ in March–April and September 2011. The average values for the CPD and PDD sites were significantly lower than those for EA2 and EA2–EAD (Table 2). In terms of the Chl-*a* concentrations between sites (EAD, CPD, and PDD) near dams in the multiple-reservoir series, EA2 and EA2–EAD had a close relationship (*r* = 0.929, *p* < 0.01), but no significant difference was observed between the other sites (*p* > 0.05).

### 3.4. Short-Term Spatial and Temporal Distributions of Water Quality after the GSM Outbreak Event

**Figure 6:**
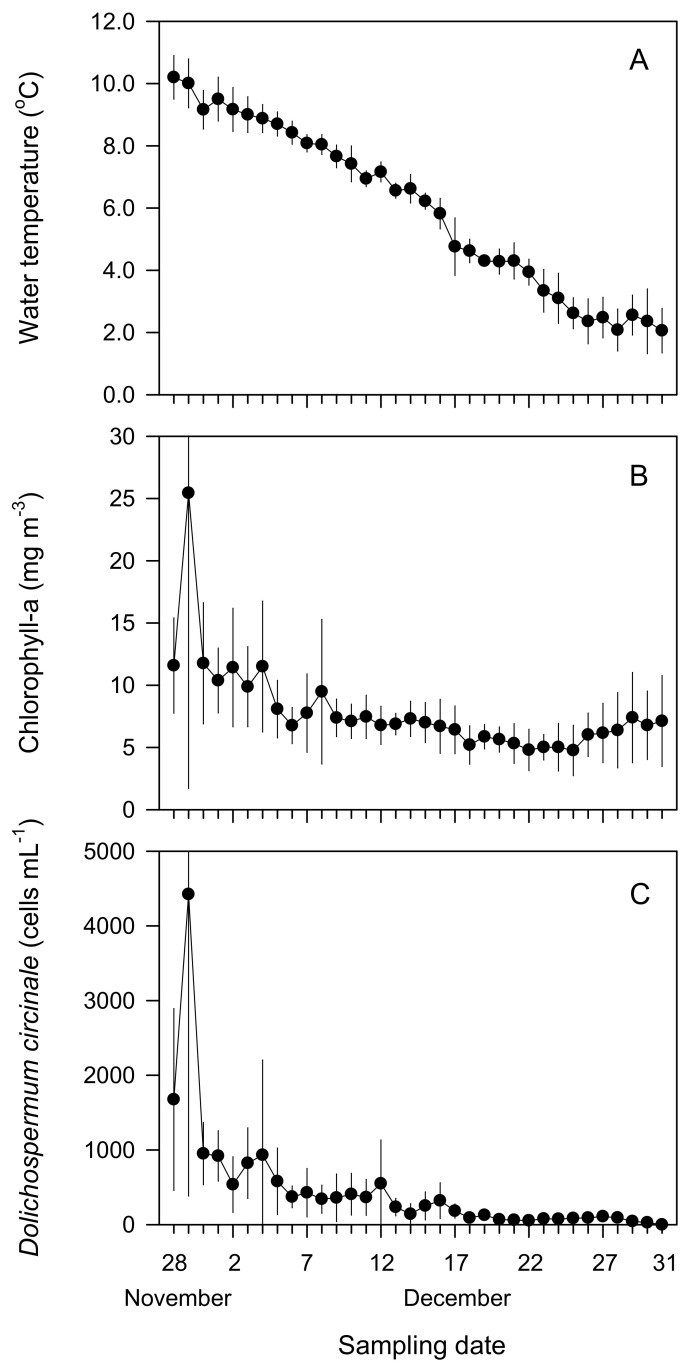
Daily variations of the (**A**) water temperatures, (**B**) chlorophyll-*a* concentrations, and (**C**) cell numbers of *Dolichospermum circinale* (formerly *Anabaena circinalis*) observed in four stations of the Euiam and Cheongpyeong Reservoirs of the North Han River and Paldang Reservoir of the Han River from 28 November to 31 December 2011.

High concentrations of GSM were detected between 14 and 24 November 2011 in the upper reaches of the Paldang Reservoir in the Han River. Table 3 summarizes the major water quality environmental factors investigated at daily intervals for approximately one month at seven sites from the EAD to the PDD after GSM was detected downstream. Figure 6 shows the daily distribution of water temperature, Chl-*a* concentration, and cell number of *D. circinale*. The water temperature (average value) ranged from 0.5 °C to 11.3 °C (5.1 °C), and most of the sites recorded temperatures below 10 °C. In addition, the gradual decrease in the water temperature over time was more remarkable (Figure 6). The Chl-*a* concentrations ranged from 1.5 to 65.0 mg m^−3^, and the average value was 9.4 mg m^−3^. At the end of November 2011, the Chl-*a* concentrations were 30.3 and 65.0 mg m^−3^ at the EAD and PDD sites, respectively, which were relatively high concentrations. At other times, all sites had concentrations <20 mg m^−3^ (Figure 6). The cell numbers of *D. circinale* in EAD and PDD at the end of November were 4576 and 11,325 cells mL^−1^, respectively, approaching or exceeding the water bloom level of 5000 cells mL^−1^ [51]. At the PD1 site, more than 3000 cells mL^−1^ were observed twice. During this period, values of >1000 cells mL^−1^ showed a high frequency (four times) at the CPD site.

### 3.5. Short-Term Spatial and Temporal Distributions of the GSM Concentration after the GSM Outbreak Event

**Figure 7:**
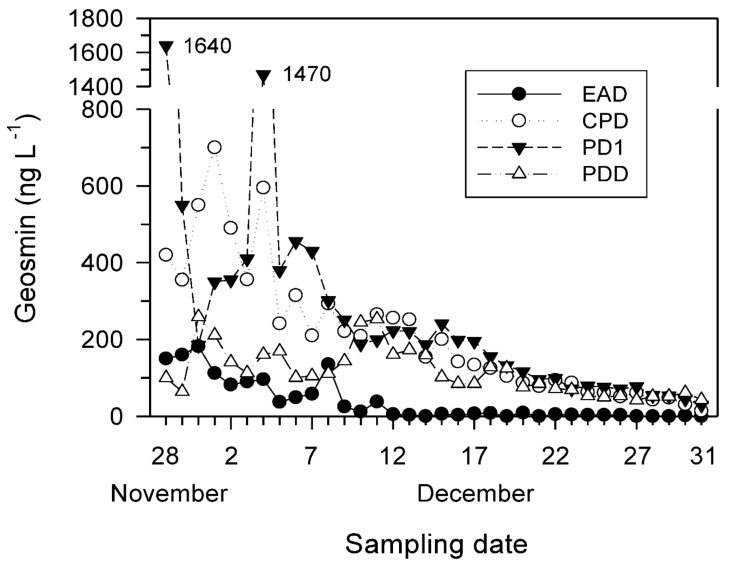
Daily variations in the geosmin concentration determined in the four stations of the Euiam and Cheongpyeong Reservoirs (EAD and CPD, respectively) of the North Han River and Paldang Reservoir (PDD) of the Han River from 28 November to 31 December 2011.

Figure 7 shows the daily variations in short-term GSM concentrations in the multiple-reservoir series from November to December 2011. GSM concentrations of >200 ng L^−1^ were observed at the CPD site (maximum 700.0 ng L^−1^) and at the PD1 (maximum 1640 ng L^−1^) and PDD sites (maximum 259.0 ng L^−1^). The GSM concentrations of >100 ng L^−1^ were maintained until mid-December (Figure 7). The EAD site had a concentration of 18.1 ng L^−1^, except at the end of November and early December 2011, which was not high compared with those of the other sites. The average values of the GSM concentration for each site were higher in the downstream than those upstream: EAD (44.0 ng L^−1^) < PDD (115.4 ng L^−1^) < CPD (198.6 ng L^−1^) < PD1 (233.2 ng L^−1^) (Figure 7 and Table 3).

## 4. Discussion

### 4.1. Hydrological and Water Quality Characteristics in a Multiple-Reservoir Series

**Figure 8:**
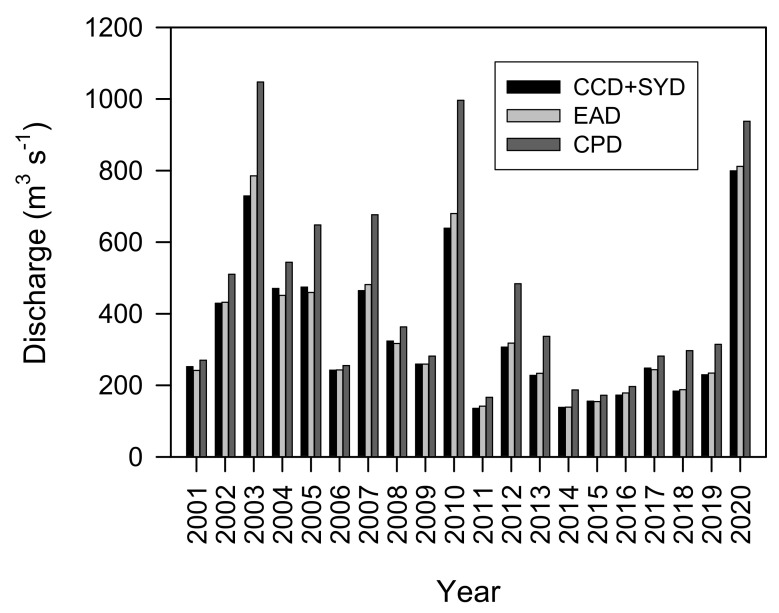
Spatial distribution of the total outflow discharged from the dams of the Chuncheon (CCD), Soyang (SYD), Euiam (EAD), and Cheongpyeong (CPD) Reservoirs between September and November every year for the past 20 years.

The water quality of the multiple-reservoir series in the water system of the North Han River featured a mutual longitudinal cause and effect based on its hydrological structure [37,40,52]. Generally, harmful cyanobacterial blooms in the long reach from the Euiam to the Paldang Reservoirs are based on the Gongji Stream estuary (EA2) and Sambong–ri (PD1) sites [37,52,53]. The severity of these blooms was greater at the EA2 site according to the pollution source structure. Even within the same water area in the Euiam Reservoir, the pattern was significantly different spatiotemporally between years or within a year [37]. From 2007 to 2012, cyanobacterial blooms were relatively severe in July 2008 (>160 × 10^3^ cells mL^−1^), August 2010, and September–November 2011 (>1000 mg Chl-*a* m^−3^). However, the bloom intensity was rather weak in 2007 and 2009 (during the opening of the sluice gate and release of the spillway for 30 d between July and September), 2011, and no major outbreaks were observed. The highest concentration (4787.0 mg Chl-*a* m^−3^) in the Euiam Reservoir in history was recorded in August 2010 (http://m.yna.co.kr (accessed on 10 August 2010)), but this was washed out by the summer rainfall (267.5 mm, 4 d) in early September. At this time, the Euiam (EAD)–Cheongpyeong (CPD)–Paldang (PDD) dam reservoirs were continuously discharged through the spillway for 10 d (<half-day to 1 d retention time) by opening the sluice gates sequentially (Figure 8). A harmful cyanobacterial bloom then broke out from late September to early November 2011. No major rainfall events occurred before, during, or after this period, and the water body dynamics and WLFs were maintained only by the hydropower-related release.

### 4.2. Source, Fate, and Effects of the Outbreak of a Harmful Cyanobacterial Bloom in an Upstream Reservoir

The persistent harmful cyanobacterial bloom that occurred in the Euiam Reservoir might be attributed to a combination of natural (meteorological factors, such as light and temperature) and anthropogenic (point or nonpoint pollution sources and hydrological fluctuations in dam operation) conditions [37,54,55,56]. Point sources with high contributions to eutrophication and harmful cyanobacterial blooms throughout the year must be highlighted [38,52]. Therefore, the frequent occurrence of harmful cyanobacterial blooms with levels of >1000 mg Chl-*a* m^−3^ up to 2011 could be attributed to the extremely high supply of P nutrients throughout the year [38,42]. This might have caused water quality problems owing to the complex action of hydrological and limnological factors of the reservoir [54,55].

Additionally, no major outbreaks of harmful cyanobacteria were observed after 2011, and odorous substances were hardly detected [41,42]. Although TP was reduced by 54.1% in the treated wastewater in 2012 and 87.6% in 2013–2014, these values were not sufficient to derive a conclusion based on the effects of point sources alone [52,57]. The threshold and sufficiently sustained concentrations to induce eutrophication and phytoplankton growth in the reservoirs were 35 and 3 µg P L^−1^, respectively [58,59]. The discharge concentrations of the treated wastewater were maintained at high levels [52], and the release of P from polluted sediment layers and internal biogenic P-load potential (e.g., recruitment) must be considered [38,42,60,61].

From the comparison of the WLFs in the three reservoirs between Euiam and Paldang from June to December 2011 (refer to Figure 3), water storage and discharge were repeated at low water levels from mid-June to early September 2011. Spillway discharges (mean values of 3103.5, 3912.6, and 6491.5 m^3^ s^−1^) from all reservoirs were performed simultaneously from late July to early August 2011. Subsequently, the range of WLFs (EL. 70.1–0.2 m) at the Euiam Reservoir was relatively small from middle to late August to early September 2011 (22 d), and the water body maintained a stable state for a long time after the rainy season flood. Consequently, the potential of harmful cyanobacterial bloom (750.8 mg Chl-*a* m^−3^) at the EA2 site was further intensified by the summer heatwave (refer to Figure 3(I)) [37]. Subsequently, the water level was gradually increased by filling the site with water for 20 d until the end of September. At this time, the flow rate of the CCD was low, and we observed that phytoplankton, which flourished in EA2, contributed significantly to the transport and dispersion of phytoplankton cells to the EAD, while simultaneously increasing the discharge of SYD and EAD (Figure 2 and Figure 3(II)) [37]. The harmful cyanobacteria transferred to the EAD were blocked by the dam and could not be delivered directly downstream (Cheongpyeong Reservoir), leading to a life cycle of growth, proliferation, and death within the Euiam reservoir [22,37,62]. This phenomenon extends from the vicinity of the dam (EAD) to upstream (CCD and SYD) depending on the time point, forming an artificial culture field in the form of an excess mat. Thus, the blooms might continue to flourish [37,38,62,63].

As soon as the water level increased, the blooms continued to decrease and increase again, and the mesoscale water level decreased in early October 2011, followed by a pulse of hydropower release (Figure 3). The low water level from middle to late October was maintained for 17 d, followed by redischarging after 5 d of filling at the end of October (Figure 3). The sudden drop in the water level at the Euiam Reservoir was due to the continuous increase in the discharge of hydroelectric power from this reservoir and decrease in the upstream dam discharges of the Chuncheon and Soyang reservoirs. At this time, unlike the overflow of the spillway owing to the opening of the water gate, the effluent was completely discharged from the middle and lower layers through the hydropower penstock [43]. All the water thus discharged downstream was transported to the CPD water area and stored for a long time during perfusion. Simultaneously, the PDD also accelerated the diffusion of water bodies by continuously increasing the discharge (Figure 3(III–IV)).

The recruitment of cyanobacteria linked to WLFs can be considered a major factor for harmful cyanobacterial outbreaks. This might occur during the water-level drawdown, decrease (e.g., discharge), and increase (e.g., impoundment) stages. This is achieved by the interaction between the water and sediment layers and is a more dominant survival strategy during water-level drawdowns and low water levels [64,65,66,67]. When the water level of a reservoir is reduced or maintained at low levels, the water bodies are vulnerable to pollution. This is because the low depth not only accelerates phytoplankton growth but also enhances the exposure of the littoral zone sediment layer, thereby promoting the recruitment of harmful cyanobacteria and release of P [60,67,68]. In addition, if spillway discharge occurs by opening the water gates, phytoplankton Chl-*a* concentrations or standing crops near the dam decrease (e.g., washout). Subsequently, regrowth occurs again at low water levels [37,40,67]. In contrast, when the water level increases, the subsequent effect of cyanobacteria recruitment due to gradual inundation of sedimentary layers can be visualized [66]. This mechanism can be considered a limnological and ecological phenomenon commonly observed in artificial dam reservoirs or weirs [29,55,69].

**Figure 9:**
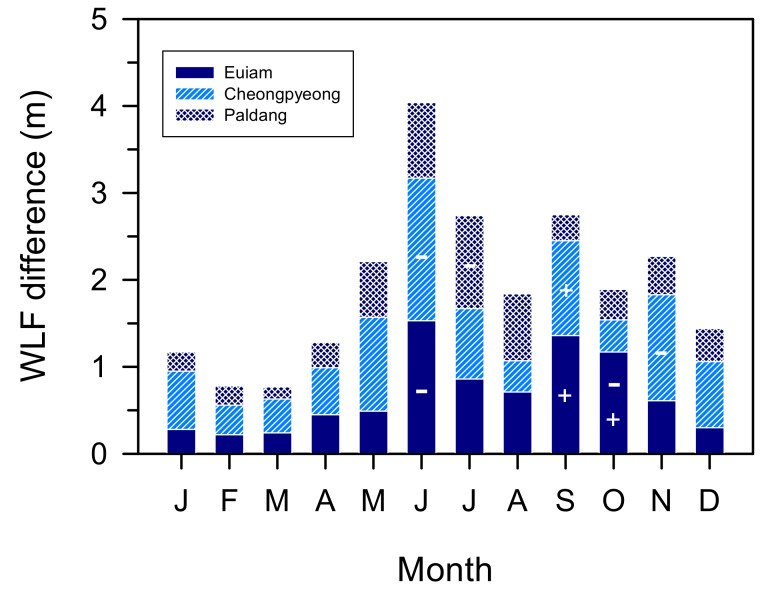
Spatial and temporal variability of the differences in the water-level fluctuations (WLFs) in the Euiam and Cheongpyeong Reservoirs of the North Han River and Paldang Reservoir of the Han River from January to December 2011. +, increase; −, decrease.

Sudden large increases in water levels may lead to reduced or flourishing biomass of harmful cyanobacteria (or other taxa), whereas significant declines in water levels may lead to undesirable phytoplankton dominance with increased biomass [67,70]. When the water level was higher than +2 m month^−1^, the cyanobacterial biomass decreased. Conversely, when the water level was below −2 m month^−1^, cyanobacterial biomass increased. If the water level is between −2 and +2 m month^−1^, cyanobacterial biomass may increase and decrease simultaneously [67]. In the water area between the Euiam and Paldang Reservoirs, the WLFs ranged from 0.14 to 1.64 m month^−1^ (average value for each reservoir: 0.47–0.77 m month^−1^), and a spatially complex variability was expected (Figure 9). From a seasonal perspective, the Chl-*a* concentration was high only at the EA2 site in June, when the water level was decreased to prepare for the monsoon flood (Figure 5B). Furthermore, the high water temperature and small and relatively stable WLFs after rainfall in August can be considered as the optimum conditions for the outbreak of harmful cyanobacteria. A combination of structural fragility (e.g., tributary confluence and dam clogging) [7] and water body non-dynamics (e.g., backflow, high retention, and particle accumulation) [7,67] at the EA2 and EAD sites occurred within the reservoir in September due to increased water-level events. This was the basis for the formation of a thick floating mat of filamentous cyanobacteria and amplification of its long-term survival (e.g., extended to late autumn or early winter) and subsequent adverse effects (e.g., water quality incident with off-flavors).

The increase in the GSM concentration in the reservoir was caused by various physical (e.g., hydraulics, hydrology, water temperature, mixing depth (*Z*_m_), and euphotic layer (*Z*_eu_)/mixing depth (*Z*_m_) ratio), chemical (e.g., DO and nutrients (ratio)), and biological (e.g., Chl-*a* concentration and density of actinomycetes) factors and had complex spatiotemporal patterns [71,72]. However, the release of off-flavor substances (e.g., GSM) from the causative cyanobacteria is caused by cell senescence or biodegradation [7,66,73] and environmental stress (e.g., feeding on zooplankton) [74,75]. The outbreak of harmful cyanobacteria in the Euiam Reservoir increased and multiplied at the source (EA2) because of the low water level, water body stability, and high nutrient pulses, and was transferred and diffused to the dam (EAD) by flow and WLFs. In addition, physical drivers facilitating cyanobacterial blooms have been demonstrated in another riverine ecosystem. In the Maumee River (a major Great Lakes tributary), low discharge and low water levels supported high retention conditions in the river estuary, in combination with low seiches. These flow-related parameters are strongly correlated with the Maumee River estuary bloom [76,77]. Subsequently, maximal growth is maintained through isolation and accumulation (reverse expansion) in the epilimnion layer [37,66]. Consequently, the thick algal mass (scum mat) died under the influence of seasonal factors, such as decreased water temperature. Off-flavor substances were subsequently excreted [7,66,71,78] and delivered downstream of the linkage dam by the hydropower release of the middle and low layers. At this time, the gradient of the concentrations of odorous substances and density of leaking algal cells could reflect the effect of the temporal patterns of hydropower discharge [43,55]. This could be identified directly or indirectly from the daily concentration distribution (Figure 5), in which the GSM concentration increased and decreased irregularly at intervals of approximately 3–4 d in the downstream reservoir.

The higher concentration at the PD1 site of the downstream reservoir compared with that of the upstream reservoir in November 2011 suggests that the odorant had already reached the Paldang Reservoir through the hydropower effluent of the upstream dam (EAD and CPD) reservoirs. The concentrations in the CPD site also increased or decreased in the range of 355.0–700.0 ng L^−1^ in early December 2011 (Figure 7). Standing crops of *D. circinale* were abundant in EAD and PDD, but the GSM concentration was low (*p* > 0.05). In contrast, CPD (*r* = 0.667, *p* < 0.01) and PD1 (*r* = 0.879, *p* < 0.01) showed the opposite pattern (Figure 5). The cell density and GSM had a coincident relationship, simultaneously increasing and decreasing or slightly preceding or following each other [20].

The GSM concentration showed a significant positive correlation with water temperature (*r* = 0.499–0.876, *p* < 0.05) and Chl-*a* concentration (*r* = 0.584–0.782, *p* < 0.01) from the Euiam to Paldang Reservoirs. In contrast, the GSM concentration in the PDD site was relatively low or not statistically significant (*p* > 0.05). The concentrations at all sites mentioned in this study were based on the main water quality standards (<10 ng L^−1^ in Japan [19] and <20 ng L^−1^ in South Korea [79]) applied at very high levels. Water treatments to render this water domestically usable requires cost (e.g., KRW 1.79 million (USD 1790) worth of powdered activated carbon (PAC) m^−3^) and effort (e.g., for at least >1–2 months) [79]. In fact, the amount of PAC used and duration in response to the water quality incident of off-the-shelf substances that occurred between November 2011 and January 2012 were approximately 760 m^3^ and 50 d, respectively. Thus, GSM can aggravate the socioeconomic damage.

### 4.3. Fate and Transport of the Cyanobacterial Bloom and GSM in a Reservoir Cascade and Management Implications

**Figure 10:**
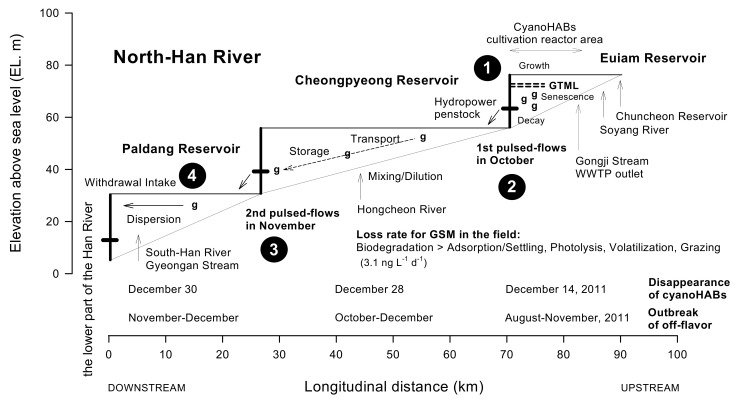
A simplified conceptual diagram illustrating the fate and transport mechanisms of the cyanobacterial bloom (Korean synonym: green tide) and geosmin that occurred in a reservoir cascade (Euiam, Cheongpyeong, and Paldang Reservoirs) from late August to late December 2011. GTML, green tide (cyanoHAB) mat layer; g, geosmin (GSM); WWTP, wastewater treatment plant; cyanoHABs, bloom-forming harmful cyanobacteria. ❶, occurrence and senescence of cyanoHABs by *Dolichospermum circinale* in the Euiam Reservoir; ❷ and ❸, first and second hypolimnetic water release through the hydropower penstock of each reservoir, respectively; ❹, outbreak of an off–flavor event in the Paldang Reservoir. The loss rate by biodegradation and other components was adopted from Graham et al. [7], Suffet et al. [19], and van Breemen et al. [25].

Based on the results of this study, we attempted to conceptually summarize the fate and transport processes of cyanobacterial blooms and their resultant GSM (Figure 10).

The widespread occurrence of harmful cyanobacterial blooms in the Euiam Reservoir was linked from the source (EA2) to the vicinity of the dam (EAD). The water that flowed to the lower part of the reservoir was blocked by the dam. Moreover, as it flowed back with a time lag, the water accumulated in the upper layer in the form of a large amount of underwater algal scum [7,37,40,55]. Over time, cells in the deeper area maintained a state in which senescence prevailed over growth due to the shortage of oxygen and light [80]. GSM was then released into the water column depending on the extent of harmful cyanobacteria occurrence (Figure 10) [18,81]. Cellular GSM release by healthy cyanobacteria in the growth phase at optimum temperatures (25–30 °C) may be low, and most of it is distributed in cells [7,63]. Only <1% of the total amount is excreted from the cell, and high concentrations are primarily observed in the subsequent lag phase and anaerobic conditions [81]. The GSM concentration in the exponential growth phase was highly correlated with the number of causative filamentous cyanobacteria (e.g., *Dolichospermum*) (*r*^2^ = 0.96) and Chl-*a* concentration (*r*^2^ = 0.95) [18,82]. The values of the GSM content per unit cell during the exponential growth and stationary phases were 8.0 and 2.5 pg, respectively, and GSM excretion reached up to 12,000 ng L^−1^ due to cell senescence in the stationary phase [18].

To determine the decrease and fate of the GSM released into the water body of a reservoir, the water is subjected to various phenomena, such as adsorption, sedimentation, photolysis, volatilization, animal feeding, biodegradation, dispersion, and dilution [25,83]. The GSM between the Euiam and Paldang Reservoirs was most likely to be delivered to the downstream reservoir after the rainy season and introduced into the hydropower effluent, which was the only transport mechanism [43,84]. The Paldang Reservoir and lower part of the Han River were eventually affected, where water intake for drinking water treatment occurred (Figure 7; http://m.chosun.com (accessed on 9 December 2011)) [53]. If the continuous discharge (e.g., pulsed flow) from the upstream reservoirs (EAD and CPD) of the North Han River was maintained, the time for the GSM material to reach the downstream reservoir (PDD) could be reduced, and the adverse impacts on the water quality could be increased [40,78]. The GSM concentration was higher in the reservoirs (5–7100 ng L^−1^) and shallow water than in the streams (4–24 ng L^−1^), with a high frequency and extent of the *Dolichospermum* bloom (Appendix A). Generally, the GSM concentrations in the reservoirs decreased (60–70% of influent water) primarily because of adsorption, sedimentation, and biodegradation (3.1 ng L^−1^ d^−1^), as described in a previous study [25]. However, if internal productivity is high (e.g., dense bloom) because of aggravated anthropogenic pollution factors, efforts to improve water quality may have adverse effects, which is intensified in eutrophic water bodies with shallow water depths [25]. In addition, GSM in stratified reservoirs with discharge systems in deep layers (middle or bottom) are mostly concentrated just below these layers, and effluents after destratification by turnover can hinder water use for several months [83].

Although biodegradation is more probable than other mechanisms (such as volatilization, photolysis, adsorption, and animal feeding) for reducing off-flavor substances in the reservoir, GSM has a relatively long half-life [83]. This indicates that preventive measures are most important for avoiding the occurrence of high-concentration GSM. In addition, predicting GSM concentrations using only simple trophic states and reservoir water quality models is very difficult [85]. This is because the average GSM concentration is the highest when the concentration of nutrients and Chl-*a* in the water is the lowest [83]. Moreover, the occurrence of GSM coincides with the time when physicochemical reactions and microbial decomposition activity rapidly weakens at the start of low water temperatures in winter. Therefore, the restriction of algal growth by the PO_4_ depletion level may be used as a determinant of GSM production potential [20].

The outbreak of harmful cyanobacteria and abnormal water quality due to GSM affected the downstream reservoirs sequentially for a prolonged period, as structural and nonstructural complex factors were interlocked (Figure 3, Figure 4 and Figure 5 and Figure 10). Nonetheless, consideration of only structural factors, such as river width and depth change, flow, water-level variability, and discharge and mixing, may be insufficient to completely understand the fate of GSM [37,40,86,87]. Basic field information, including the dynamics of major pollutants, life history, and recruitment of harmful cyanobacteria, may also be crucial [37,41,53,88]. Finally, we emphasize the real-time monitoring of treated wastewater and hydropower effluent to avoid water quality incidents related to the transport and dispersion of harmful cyanobacterial blooms and GSM in the environment of a multi-reservoir series.

## 5. Conclusions

In the current study, we analyzed the relationship between the harmful cyanobacterial outbreak and the resultant production and transport of GSM in a multi-reservoir series. We conclude that the worst water quality incident and its prolonged persistence in the downstream reservoirs were caused by meteorohydrological factors (e.g., cold water, water-level drawdown, and pulsed effluents by hydropower operation). However, we also suggest that spatiotemporal variation in water quality parameters be considered, which could provide crucial information to improve the understanding of year-round dynamics of cyanobacterial blooms and GSM. Cyanobacterial blooms and the resultant harmful substances (e.g., toxins and off-flavor material) in a reservoir cascade can be an important problem, considering their downstream transport. Therefore, various subsequent impacts on aquatic ecosystems and water use are challenges that must be investigated in future studies.

## Figures and Tables

**Table 1:** Descriptive statistics of the hydrological factors determined in six reservoirs located on the North Han River and Han River systems from January to December 2011. The values indicate means, standard deviations, and ranges (minimum and maximum). The results of Tukey’s test are represented by ^a^–^f^, where stations with different letters are statistically different from each other (^a^ > ^b^ > ^c^ > ^d^ > ^e^ > ^f^) and stations with the same letters are statistically similar.

Factors\Reservoirs	Hwacheon	Chuncheon	Soyang	Euiam	Cheongpyeong	Paldang	*p*
Daily rainfall (mm)	-	-	9.7 ± 19.9 (0.1–155.4)	-	-	-	-
Total inflow (m^3^ s^−1^)	86.9 ± 267.5 ^a^ (0.0–2814.4)	128.6 ± 338.4 ^a,b^ (0.0–3721.7)	105.5 ± 291.7 ^a^ (0.0–3042.2)	252.7 ± 565.2 ^a,b,c^ (23.5–6442.2)	329.0 ± 776.3 ^c^ (5.9–8757.9)	877.4 ± 1670.1 ^d^ (60.0–13239.6)	0.001
Total outflow (m^3^ s^−1^)	92.1 ± 242.3 ^a^ (0.0–2492.6)	128.6 ± 339.8 ^a^ (0.0–3839.0)	105.9 ± 170.2 ^a^ (1.9–1513.7)	252.7 ± 566.1 ^a,b^ (0.0–6482.1)	328.7 ± 777.3 ^b^ (0.0–8710.3)	877.6 ± 1670.6 ^c^ (124.5–13,317.4)	0.001
Spillway outflow (m^3^ s^−1^)	-	-	886.4 ± 462.8 (32.8–1318.2)	-	-	-	-
Hydropower release (m^3^ s^−1^)	-	-	85.3 ± 57.2 (0.0–232.7)	-	-	-	-
Water level (EL.m)	170.3 ± 3.5 ^e^ (158.7–178.5)	102.2 ± 0.5 ^d^ (100.6–102.7)	179.6 ± 7.3 ^f^ (164.3–193.3)	70.7 ± 0.5 ^c^ (69.6–71.3)	50.2 ± 0.4 ^b^ (48.9–50.9)	25.1 ± 0.2 ^a^ (24.3–25.4)	0.001
Annual WLF (m)	19.8	2.1	29.0	1.7	2.0	1.1	-

WLF, water-level fluctuation.

**Table 2:** Descriptive statistics of the monthly water quality factors determined for the major stations of the three reservoirs located on the North Han River and Han River basins from January to December 2011. The values indicate means, standard deviations, and ranges (minimum and maximum). The results of Tukey’s test are represented by ^a^–^c^, where stations with different letters are statistically different from each other (^a^ > ^b^ > ^c^) and stations with the same letters are statistically similar. Data were obtained from the Korea Water Environmental Information System (http://water.nier.go.kr (accessed on 1 April 2012)) and from two stations (EA2 and EAD) additionally surveyed on 13 August and 19 September 2011. Refer to Figure 1 for the locations of the different stations.

Factors/Stations	Euiam Reservoir			Cheongpyeong Reservoir		Paldang Reservoir				*p*
	EA1	EA2	EAD	CP1	CPD	PD1	PD2	PD3	PDD	
Temperature (°C)	14.6 ± 5.5 (6.0–22.0)	14.0 ± 5.5 (7.0–23.0)	12.2 ± 6.4 (3.0–20.4)	14.6 ± 6.6 (4.0–24.1)	15.4 ± 5.7 (5.7–23.3)	12.3 ± 7.5 (1.0–22.3)	11.8 ± 7.6 (1.0–22.0)	13.5 ± 8.9 (1.0–23.8)	12.0 ± 7.5 (1.0–22.0)	0.920
DO (mg O_2_ L^−1^)	13.2 ± 0.9 (9.5–16.7)	12.1 ± 2.7 (7.9–16.8)	12.5 ± 1.8 (8.9–14.7)	11.1 ± 2.1 (8.9–15.5)	11.1 ± 1.6 (8.8–13.6)	12.2 ± 1.8 (10.3–16.1)	12.1 ± 1.4 (10.2–14.3)	12.2 ± 2.2 (9.1–15.7)	12.1 ± 1.6 (10.1–15.3)	0.454
pH	7.8 ± 0.5 (7.0–9.0)	7.7 ± 0.7 (6.4–9.0)	7.8 ± 0.3 (7.3–8.3)	7.8 ± 0.3 (7.3–8.3)	7.8 ± 0.4 (7.3–8.7)	8.0 ± 0.4 (7.5–9.2)	7.8 ± 0.4 (7.2–8.3)	8.0 ± 0.6 (7.2–9.2)	8.0 ± 0.4 (7.5–9.1)	0.707
Conductivity (μS cm^−1^)	102.1 ± 32.1 ^a^ (66.0–149.0)	97.5 ± 30.5 ^a^ (60.0–151.0)	87.1 ± 10.3 ^a^ (74.0–109.0)	83.7 ± 13.2 ^a^ (60.0–105.0)	84.4 ± 12.1 ^a^ (71.0–105.0)	210.6 ± 34.0 ^b^ (146.0–248.0)	107.0 ± 25.2 ^a^ (74.0–149.0)	194.2 ± 59.9 ^b,c^ (107.0–300.0)	210.8 ± 32.6 ^c^ (148.0–247.0)	0.001
TSS (mg dw L^−1^)	3.5 ± 2.3 (0.4–6.9)	3.6 ± 2.5 (0.5–7.9)	5.3 ± 4.8 (1.1–18.9)	4.6 ± 2.3 (2.0–9.8)	5.3 ± 3.2 (2.2–13.0)	32.0 ± 49.3 (2.6–146.4)	4.7 ± 2.8 (2.5–11.8)	13.6 ± 23.3 (1.7–86.5)	28.6 ± 46.8 (3.3–162.6)	0.043
BOD_5_ (mg O_2_ L^−1^)	0.8 ± 0.9 ^a^ (0.2–3.2)	0.8 ± 0.5 ^a^ (0.3–1.7)	0.7 ± 0.3 ^a^ (0.2–1.3)	1.1 ± 0.5 ^a^ (0.5–1.8)	0.9 ± 0.3 ^a^ (0.5–1.2)	1.3 ± 0.9 ^a^ (0.5–3.6)	1.0 ± 0.3 ^a^ (0.5–1.5)	2.0 ± 1.1 ^b^ (1.0–4.2)	1.2 ± 0.9 ^a,b^ (0.5–3.2)	0.001
COD_Mn_ (mg O_2_ L^−1^)	3.3 ± 1.2 ^a^ (2.3–6.3)	3.0 ± 1.0 ^a^ (2.2–5.6)	3.1 ± 0.5 ^a^ (2.6–4.3)	3.1 ± 0.5 ^a^ (2.2–3.7)	2.9 ± 0.4 ^a^ (2.3–3.6)	4.2 ± 1.8 ^a,b^ (2.2–8.0)	3.3 ± 0.4 ^a,b^ (2.7–4.1)	4.5 ± 1.1 ^b^ (2.9–6.1)	4.1 ± 1.7 ^a,b^ (2.6–7.8)	0.001
T–N (μg N L^−1^)	2743.8 ± 1025.6 ^b,c^ (1417.0–4657.0)	2976.3 ± 913.9 ^c^ (1646.0–4284.0)	2565.4 ± 801.6 ^a,b,c^ (1610.0–3903.0)	1775.5 ± 384.0 ^a^ (1383.0–2656.0)	1788.3 ± 319.7 ^a^ (1494.0–2464.0)	2759.8 ± 355.0 ^a,b,c^ (2284.0–3503.0)	1919.7 ± 260.8 ^a,b^ (1615.0–2503.0)	2794.8 ± 740.7 ^c^ (1783.0–3995.0)	2731.7 ± 334.5 ^b,c^ (2339.0–3231.0)	0.001
DTN (μg N L^−1^)	2118.4 ± 765.7 ^a,b,c^ (1323.0–3494.0)	2247.3 ± 764.4 ^a,b,c^ (1,372.0–3,406.0)	1951.3 ± 553.9 ^a,b^ (1401.0–3452.0)	1708.0 ± 359.1 ^a^ (1367.0–2540.0)	1731.3 ± 303.4 ^a^ (1385.0–2362.0)	2660.2 ± 359.1 ^a,b,c^ (2209.0–3454.0)	1868.8 ± 259.3 ^a^ (1575.0–2445.0)	2687.3 ± 687.8 ^c^ (1751.0–3774.0)	2640.5 ± 341.6 ^b,c^ (2173.0–3146.0)	0.001
Ammonium (μg N L^−1^)	103.2 ± 163.5 (0.0–467.0)	40.0 ± 43.8 (0.0–107.0)	54.6 ± 30.8 (21.0–114.0)	22.6 ± 10.9 (6.0–38.0)	19.5 ± 9.7 (8.0–41.0)	66.9 ± 61.8 (15.0–195.0)	32.2 ± 20.2 (8.0–80.0)	72.5 ± 44.9 (12.0–157.0)	86.2 ± 99.1 (19.0–330.0)	0.050
Nitrate (μg N L^−1^)	1399.8 ± 544.8 ^a^ (125.0–1907.0)	1413.8 ± 576.5 ^a^ (233.0–2478.0)	1489.5 ± 1508.0 ^a^ (1120.0–1962.0)	1423.7 ± 279.9 ^a^ (843.0–1864.0)	1443.5 ± 214.2 ^a^ (1192.0–1836.0)	2,151.5 ± 312.3 ^a,b^ (1644.0–2677.0)	1521.3 ± 280.8 ^a^ (1261.0–2127.0)	2120.2 ± 660.5 ^b^ (1358.0–3461.0)	2133.7 ± 290.8 ^b^ (1719.0–2607.0)	0.001
T–P (μg P L^−1^)	38.1 ± 41.6 ^a,b^ (5.0–145.0)	48.3 ± 34.3 ^a,b^ (18.0–123.0)	42.8 ± 27.3 ^a,b^ (20.0–99.0)	25.7 ± 10.2 ^a^ (8.0–44.0)	39.4 ± 24.6 ^a,b^ (14.0–77.0)	67.7 ± 44.2 ^a,b^ (33.0–161.0)	24.4 ± 9.7 ^a^ (14.0–43.0)	49.8 ± 24.8 ^a,b^ (13.0–100.0)	70.8 ± 51.7 ^b^ (34.0–183.0)	0.022
DTP (μg P L^−1^)	21.1 ± 29.4 (1.0–91.0)	23.3 ± 15.8 (7.0–56.0)	24.0 ± 19.9 (8.0–81.0)	22.5 ± 9.4 (4.0–35.0)	32.6 ± 22.7 (11.0–72.0)	29.9 ± 12.9 (13.0–55.0)	12.1 ± 5.4 (5.0–21.0)	23.8 ± 12.4 (5.0–44.0)	31.7 ± 14.7 (13.0–57.0)	0.167
SRP (μg P L^−1^)	14.3 ± 27.9 ^a,b^ (0.0–84.0)	6.4 ± 5.9 ^a,b^ (0.0–20.0)	5.9 ± 2.1 ^a,b^ (2.0–9.0)	8.0 ± 5.2 ^a,b^ (2.0–19.0)	14.3 ± 15.7 ^a,b^ (3.0–48.0)	19.7 ± 14.7 ^a,b^ (2.0–47.0)	3.8 ± 3.7 ^a^ (0.0–12.0)	12.3 ± 12.7 ^a,b^ (2.0–35.0)	21.4 ± 13.5 ^b^ (1.0–46.0)	0.030
Chl-*a* (mg m^−3^)	5.5 ± 7.9 (0.1–20.9)	154.3 ± 328.3 (0.5–950.8)	10.5 ± 10.6 (2.1–35.3)	6.9 ± 4.5 (1.3–15.5)	6.3 ± 3.7 (1.3–11.3)	14.0 ± 13.6 (1.8–40.8)	11.0 ± 5.8 (1.9–19.6)	16.2 ± 9.4 (4.7–34.4)	10.3 ± 9.7 (1.9–33.7)	0.166

DO, dissolved oxygen; TSS, total suspended solids; BOD_5_, biological oxygen demand; COD_Mn_, chemical oxygen demand; TN, total nitrogen; DTN, dissolved total nitrogen; TP, total phosphorus; DTP, dissolved total phosphorus; SRP, soluble reactive phosphorus; Chl-*a*, chlorophyll-*a*.

**Table 3:** Descriptive statistics of the daily water quality factors determined in the major stations of three reservoirs in the North Han River and Han River basins from 28 November 2011 to 4 January 2012. The values indicate means, standard deviations, and ranges (minimum and maximum). The results of Tukey’s test are represented by ^a^–^f^, where stations with different letters are statistically different from each other (^a^ > ^b^ > ^c^ > ^d^ > ^e^ > ^f^) and stations that share the same letters are statistically similar. Data were obtained from the Ministry of Environment (MOE) and Korea Water Resources Corporation. Refer to Figure 1 for the locations of the different monitoring stations.

Factors/Stations	Euiam Reservoir	Cheongpyeong Reservoir		Paldang Reservoir				*p*
	EAD	CP1	CPD	PD1	PD2	PD3	PDD	
Temperature (°C)	6.1 ± 2.4 ^b^ (2.6–9.8)	5.3 ± 2.5 ^b^ (1.6–9.7)	6.2 ± 2.7 ^b^ (2.3–11.3)	5.7 ± 2.9 ^b^ (1.3–10.7)	4.9 ± 3.0 ^b^ (0.5–9.6)	1.6 ± 0.5 ^a^ (1.1–2.6)	5.6 ± 2.9 ^b^ (1.1–10.3)	0.001
Conductivity (μS cm^−1^)	83.8 ± 6.9 ^a^ (72.0–101.0)	83.8 ± 5.2 ^a^ (76.0–99.0)	93.6 ± 8.1 ^a,b^ (80.0–107.0)	105.1 ± 8.3 ^b^ (97.0–136.0)	230.6 ± 14.0 ^d^ (199.0–268.0)	283.3 ± 29.7 ^e^ (217.0–320.0)	172.7 ± 21.9 ^c^ (130.0–211.0)	0.001
T–N (μg N L^−1^)	1887.1 ± 201.2 ^a,b^ (1600.0–2300.0)	1835.7 ± 144.6 ^a^ (1600.0–2300.0)	2041.9 ± 189.3 ^b,c^ (1800.0–2500.0)	2196.6 ± 195.5 ^c^ (1800.0–2700.0)	2945.2 ± 244.7 ^e^ (2200.0–3300.0)	4368.4 ± 547.8 ^f^ (3400.0–5600.0)	2554.8 ± 207.9 ^d^ (2000.0–2800.0)	0.001
T–P (μg P L^−1^)	29.5 ± 7.4 ^a^ (17.0–45.0)	26.0 ± 6.3 ^a^ (17.0–45.0)	21.5 ± 3.4 ^a^ (16.0–29.0)	22.0 ± 3.3 ^a^ (17.0–28.0)	50.8 ± 27.4 ^b^ (29.0–100.0)	57.2 ± 30.1 ^b^ (31.0–100.0)	33.5 ± 13.9 ^a^ (20.0–100.0)	0.001
SRP (μg P L^−1^)	5.0 ± 4.1 ^a,b^ (0.0–16.0)	3.3 ± 2.9 ^a,b^ (0.0–13.0)	0.9 ± 0.9 ^a^ (0.0–3.0)	1.6 ± 1.6 ^a^ (0.0–6.0)	19.8 ± 9.1 ^d^ (7.0–42.0)	11.5 ± 14.8 ^c^ (0.0–46.0)	7.1 ± 3.8 ^b,c^ (2.0–16.0)	0.001
Chl-*a* (mg m^−3^)	8.3 ± 6.0 ^b^ (2.1–30.3)	7.1 ± 3.3 ^a,b^ (3.1–16.6)	8.4 ± 2.0 ^b^ (4.5–13.0)	6.7 ± 2.0 ^a,b^ (4.5–12.4)	3.6 ± 1.8 ^a^ (1.5–11.4)	23.7 ± 10.2 ^c^ (10.9–38.7)	8.0 ± 10.4 ^a,b^ (4.4–65.0)	0.001
Algal standing crops (cells mL^−1^)	1652 ± 202 ^a^ (610–6616)	1266 ± 547 ^a^ (640–3186)	1402 ± 832 ^a^ (616–4344)	1782 ± 1338 ^a^ (582–6480)	1,069 ± 359 ^a^ (670–2061)	9601 ± 5925 ^b^ (1,291–20,267)	2017 ± 2600 ^a^ (692–15665)	0.001
Cyanobacterial density (cells mL^−1^)	696 ± 1210 (21–4576)	449 ± 450 (47–1466)	459 ± 474 (36–1874)	627 ± 927 (27–3574)	110 ± 46 (59–150)	58 ± 13 (49–67)	898 ± 2288 (52–11325)	0.859
Geosmin (ng L^−1^)	44.0 ± 57.7 ^a^ (2.0–182.0)	125.0 (only)	198.6 ± 163.6 ^b,c^ (14.0–700.0)	233.2 ± 295.9 ^c^ (29.0–1640.0)	10.1 ± 17.7 ^a^ (2.0–68.0)	61.0 ± 17.5 ^a,b^ (38.0–91.0)	115.4 ± 61.0 ^a,b,c^ (42.0–259.0)	0.001

TN, total nitrogen; TP, total phosphorus; SRP, soluble reactive phosphorus; Chl-*a*, chlorophyll-*a*.

## Supplementary Materials

The following supporting information can be downloaded at: https://www.mdpi.com/article/10.3390/ijerph19159294/s1, Table S1: General geographic and limnological features of hydropower and multipurpose dam reservoirs located in the North-Han and the Han Rivers system.

## Appendix A

**Table A1:** Comparison of geosmin distribution with the occurrence of bloom-forming cyanobacteria in different aquatic ecosystems.

Country	Aquatic Ecosystems	Geosmin (ng L^−1^)	Dominant Species	Chlorophyll-*a* (mg m^−3^)	Standing crops (cells mL^−1^)	References
America	RI (St. Lawrence)	5–20	Periphyton			[23]
	RE (Diamond Valley)	750	*Ana. circinalis*	–	–	[62]
Australia		4000 (0.01 pg cell^−1^)	–	–	–	[8]
China	RE (Yanghe)	>7000	*Ana. spiroides*	–	–	[89]
		7100 (0.1 pg cell^−1^)	*Ana. spiroides*	–	–	[9]
Japan	RE (Biwa)	1050	*–*	–	–	[90]
South Africa		3170	–	–	–	[11]
Korea	RE (Paldang)	1470–1640	*Ana. spiroides*	–	3185–3574	[53]
		385	*Ana. circinalis*	–	2280	[91]
	RI (Geum)	32–161	*–*	–	–	[92]
	RE (Daecheong)	5.3	*Ana. spiroides*	24.5	17,000	[93]
		11–303	*–*	–	–	[92]
		21–963	*Anabaena* sp. *Aphanizomenon* sp. *Oscillatoria* sp.	–	–	[94,95]
	RI (Nakdong)	4–24	*Microcystis* sp. *Aphanizomenon* sp.	–	–	[96]
	RE (Dongbok)	171	*Ana. macrospora*	30.5	373.7 (23,400 colony)	[97]

RI, river; RE, reservoir; Ana., *Anabaena*.
