# Supplementary material for: Downstream Transport of Geosmin Based on Harmful Cyanobacterial Outbreak Upstream in a Reservoir Cascade
Source: Int J Environ Res Public Health. 2022 Jul 29;19(15):9294. doi: 10.3390/ijerph19159294 (PMC9368543; doi:10.3390/ijerph19159294)
Supplement: Supplementary file 1 [file ijerph-19-09294-s001.zip › ijerph-1824531-supplementary.pdf]

Supplementary Table S1. General geographic and limnological features of hydropower and multipurpose dam reservoirs located in the North-Han and the Han Rivers system.

Attribute	Hwacheon	Chuncheon	Soyang	Euiam	Cheongpyeong	Paldang
River system	North-Han	North-Han	Soyang	North-Han	North-Han	Han
Elevation (EL.m)	183.0	104.9	198.0	73.3	52.0	27.0
Longitudinal distance (km)	122.9	89.7	80.0	70.3	26.5	0.0
Watershed area (km^2)	3901	4736	2703	7709	9921	23800
Yearly average precipitation (mm)	1137.0	1161.0	1100.0	1129.1	1165.3	1159.9
Yearly average inflow ($m^3 s^{-1}$)	92.8	111.4	55.5	185.2	229.7	538.5
Yearly average outflow ($m^3 s^{-1}$)	-	-	-	-	-	-
Dam hight (m)	81.5	40.0	123.0	23.0	31.0	29.0
Dam length (m)	435.0	453.0	530.0	273.0	407.0	575.0
Dam type	CG	CG	ER	CG	CG	CG
Flood water level (EL.m, FWL)	183.0	104.9	198.0	73.8	52.0	27.0
Normal high water level (EL.m, NHWL)	181.0	103.0	193.5	71.5	51.0	25.5
Restricted water level (EL.m, RWL)	175.0	102.0	190.3	70.5	50.0	-
Low water level (EL.m, LWL)	156.8	98.2	150.0	66.3	46.0	25.0
Penstock level (EL.m)	103.0	74.1	78.5	55.2	27.1	13.5
Total impoundment (10^6 m^3)	1018.0	150.0	2900.0	80.0	185.5	244.0
Efficiency impoundment (10^6 m^3)	658.0	61.0	1900.0	57.5	82.6	18.0
Flood control quantity (10^6 m^3)	213.0	-	500.0	-	-	-
Reservoir area (km^2)	38.2	14.3	70.0	15.5	17.6	36.5
Watergate type and numbers	Roller, 16	Tainter, 12	Tainter, 5	Roller, 14	Roller, 24	Tainter, 15
Hydropower generation (GWh yr^{-1})	326	145	353	161	272	378
Construction year	1944	1965	1973	1967	1943 (~1968)	1974

CG: concrete gravity, ER: rock-fill embankment.
